# Birth “Out-of-Hours”: An Evaluation of Obstetric Practice and Outcome According to the Presence of Senior Obstetricians on the Labour Ward

**DOI:** 10.1371/journal.pmed.1002000

**Published:** 2016-04-19

**Authors:** Hannah E. Knight, Jan H. van der Meulen, Ipek Gurol-Urganci, Gordon C. Smith, Amit Kiran, Steve Thornton, David Richmond, Alan Cameron, David A. Cromwell

**Affiliations:** 1 Department of Health Services Research and Policy, London School of Hygiene and Tropical Medicine, London, United Kingdom; 2 Lindsay Stewart Centre for Audit and Clinical Informatics, Royal College of Obstetricians and Gynaecologists, London, United Kingdom; 3 Department of Obstetrics and Gynaecology, University of Cambridge, Cambridge, United Kingdom; 4 Department of Obstetrics and Gynaecology, University of Exeter Medical School, Exeter, United Kingdom; University of Manchester, UNITED KINGDOM

## Abstract

**Background:**

Concerns have been raised that a lack of senior obstetricians (“consultants”) on the labour ward outside normal hours may lead to worse outcomes among babies born during periods of reduced cover.

**Methods and Findings:**

We carried out a multicentre cohort study using data from 19 obstetric units in the United Kingdom between 1 April 2012 and 31 March 2013 to examine whether rates of obstetric intervention and outcome change “out-of-hours,” i.e., when consultants are not providing dedicated, on-site labour ward cover.

At the 19 hospitals, obstetric rotas ranged from 51 to 106 h of on-site labour ward cover per week. There were 87,501 singleton live births during the year, and 55.8% occurred out-of-hours. Women who delivered out-of-hours had slightly lower rates of intrapartum caesarean section (CS) (12.7% versus 13.4%, adjusted odds ratio [OR] 0.94; 95% confidence interval [CI] 0.90 to 0.98) and instrumental delivery (15.6% versus 17.0%, adj. OR 0.92; 95% CI 0.89 to 0.96) than women who delivered at times of on-site labour ward cover. There was some evidence that the severe perineal tear rate was reduced in out-of-hours vaginal deliveries (3.3% versus 3.6%, adj. OR 0.92; 95% CI 0.85 to 1.00). There was no evidence of a statistically significant difference between out-of-hours and “in-hours” deliveries in the rate of babies with a low Apgar score at 5 min (1.33% versus 1.25%, adjusted OR 1.07; 95% CI 0.95 to 1.21) or low cord pH (0.94% versus 0.82%; adjusted OR 1.12; 95% CI 0.96 to 1.31). Key study limitations include the potential for bias by indication, the reliance upon an organisational measure of consultant presence, and a non-random sample of maternity units.

**Conclusions:**

There was no difference in the rate of maternal and neonatal morbidity according to the presence of consultants on the labour ward, with the possible exception of a reduced rate of severe perineal tears in out-of-hours vaginal deliveries. Fewer women had operative deliveries out-of-hours.

Taken together, the available evidence provides some reassurance that the current organisation of maternity care in the UK allows for good planning and risk management. However there is a need for more robust evidence on the quality of care afforded by different models of labour ward staffing.

## Abstract 2

In a multicenter cohort study, Hannah Knight and colleagues assess differences in birth-related outcomes associated with the presence of senior obstetricians on the labor ward.

## Editors' Summary

**Background:**

In an ideal world, expert medical care would be available for anyone who needs it 24 hours a day, 7 days a week. But in the real world, a lower level of medical cover is often available in the evening and at weekends, a situation that potentially puts lives at risk. In the United Kingdom, for example, the National Health Service (NHS) does not currently provide consultant-level care or full laboratory and imaging services in the evenings and at weekends (in the UK, a consultant is a senior, hospital-based doctor who provides care in a medical or surgical specialty). Consequently, patients admitted out of normal hours have to wait to see a consultant and to have tests and scans done. Citing studies that suggest that people admitted to hospital at the weekend are more likely to die than those admitted on weekdays, the UK government recently made a pledge to improve access to consultants and other NHS services during evenings and weekends.

**Why Was This Study Done?:**

Maternity care is a specialty in which a 24-hour hospital service is clearly required. Women can go into labor at any time of the day, and intrapartum emergencies (emergencies that arise during labor) can develop rapidly and without warning in previously uncomplicated pregnancies. Concerns have been raised that a lack of senior obstetricians (consultants who look after women during pregnancy, childbirth, and the postpartum period immediately after childbirth) on labor wards outside normal working hours may lead to worse outcomes among babies born during periods of reduced cover. However, few studies have examined the extent to which variation in consultant presence on labor wards affects maternal and neonatal (newborn) outcomes. In this multicenter cohort study, the researchers use data from UK obstetric units to evaluate the relationship between the presence of obstetric consultants on labor wards and the rates of obstetric interventions (surgical delivery by cesarean section and “instrumental” delivery using forceps or a vacuum) and of several maternal and neonatal outcomes.

**What Did the Researchers Do and Find?:**

For their study, the researchers used electronic patient data collected over a 12-month period by 19 obstetric units and administrative data on obstetric rotas at the participating hospitals, which were mainly teaching hospitals. On-site labor ward cover by consultants ranged from 51 to 106 hours per week at the participating hospitals, where there were 87,501 singleton live births over the study period, 55.8% of which occurred “out-of-hours.” Women who delivered out-of-hours had slightly lower rates of intrapartum cesarean section (operations initiated after labor had started; 12.7% versus 13.4%) and of instrumental delivery (15.6% versus 17.0%) than women who delivered when there was on-site labor ward cover (“in-hours”). Moreover, whereas 3.6% of women who had an in-hours vaginal delivery had a severe perineal tear (damage to the soft tissue between the vagina and the anus), only 3.3% of women who delivered out-of-hours had a tear. Finally, there was no difference between out-of-hours and in-hours deliveries in the rate of babies with a low Apgar score 5 minutes after birth (a measure of newborn health) or a low cord pH (a measure of oxygen deprivation during birth) or in the rate of mothers with severe postpartum bleeding.

**What Do These Findings Mean?:**

These findings show no association between maternal and neonatal outcomes and the presence of consultants on the labor ward, with the exception of weak evidence for a reduced likelihood of severe perineal tears following out-of-hours delivery. However, women who deliver out-of-hours may be less likely to have an obstetric intervention than women who deliver in-hours, possibly because obstetric teams will usually try to undertake operative deliveries during in-hours shifts. The accuracy of these findings may be limited by the use of administrative records to determine when consultants were present on labor wards and by the potential for “bias by indication.” That is, obstetric teams may have tried to ensure that women with a greater risk of a poor outcome delivered in-hours rather than out-of-hours. Nevertheless, these findings suggest that the current organization of maternity care in the UK allows for good planning and risk management. Thus, although further robust evidence on the quality of care delivered at all times of the week by UK maternity units is needed, politically driven efforts to increase senior obstetrician attendance “out-of-hours” may not lead to improved clinical outcomes for women and babies.

**Additional Information:**

This list of resources contains links that can be accessed when viewing the PDF on a device or via the online version of the article at http://dx.doi.org/10.1371/journal.pmed.1002000.

- This study is further discussed in a *PLOS Medicine* Perspective by Jenny Myers and Edward Johnstone
- Information from the UK Department of Health on research into the weekend effect on hospital mortality and the UK government's plans to provide NHS 7-day services by 2020 is available; a news article from the BBC discusses an ongoing controversy about how the UK government has used a recent research study to back its case for more 7-day NHS care
- The UK National Health Service website provides information on current out-of-hours NHS services, labor and childbirth (including a video about giving birth in hospital), cesarean section, assisted delivery (including a video), and perineal tears
- The UK Royal College of Obstetricians and Gynaecologists provides patient information leaflets on all aspects of pregnancy and childbirth
- MedlinePlus provides links to sources of other information on childbirth

## Introduction

The new United Kingdom government has made a commitment to extend access to National Health Service (NHS) services during evenings and weekends [1]. The policy focuses attention on the quality of care delivered out of normal hours and the concerns that have been raised by recent studies examining the outcomes of hospital services [2,3].

Maternity care is a prime example of when a 24-h hospital service is required–women may begin labour at any time of day, and intrapartum emergencies may develop rapidly and without warning, often in previously uncomplicated pregnancies. In recent years, several large population-based studies have produced evidence to suggest that perinatal outcomes are slightly worse among babies born outside normal office hours [4,5]. In particular, Pasupathy et al. analysed Scottish data on 1 million liveborn, term, cephalic, singleton births between 1985 and 2004 and reported a neonatal mortality rate (excluding deaths due to congenital abnormalities) of 0.42 per 1,000 between 09:00 and 17:00 on Monday to Friday and a rate of 0.56 per 1,000 outside of this time [4].

Pasupathy et al. postulated that their findings could be related to variation in staffing at different times of day [4]. The impact of different models of labour ward staffing on perinatal outcomes has been part of a continuing debate about the delivery of maternity care in several countries, with investigations into poor-quality care and adverse events regularly highlighting concerns about inadequate staffing levels [6,7]. One aspect of this debate has been on the lack of senior obstetricians (“consultants”) on the labour ward outside normal hours and the potential benefits of 24-h-per-day consultant cover for both quality of care and the training and supervision of junior doctors [7–11]. In the UK, the Royal College of Obstetricians and Gynaecologists (RCOG) supports a 24-h-per-day, consultant obstetrician-led service but recognises that its implementation poses many challenges in terms of job plans, remuneration, and labour ward facilities [11]. Currently, the number of hours and pattern of consultant presence over the week varies widely among UK maternity units [12]. Clinical standards first published by the RCOG in 2007, and reiterated again in 2011 [13], recommend a minimum of two consultant-led ward rounds (i.e., with the consultant physically present) on Saturdays, Sundays, and bank holidays, and one during the evening [11].

Few studies have examined the extent to which variation in consultant presence on the labour ward contributes to maternal and neonatal outcomes. Woods et al. found no association between consultant presence and mode of delivery or low Apgar score at 1 and 5 min, but the study was limited to 20,187 deliveries in a single UK obstetric unit [14]. Likewise, Ahmed et al. found no objective evidence of the benefits of introducing resident 24/7 consultant cover on patient care in a single tertiary maternity unit [15].

In this study, we investigated whether obstetric practice and outcome varied according to the presence of obstetric consultants on the labour ward using a large clinical dataset of deliveries at 19 UK obstetric units during 2012–13. The study evaluated the relationship between consultant presence and three neonatal outcomes: Apgar score < 7 at 5 min; umbilical cord pH less than 7.1, and admission to neonatal care. In addition, we examined the relationship between consultant presence and operative deliveries (instrumental or intrapartum CS) and severe maternal outcomes (third or fourth degree perineal tear and severe postpartum haemorrhage [PPH]. To our knowledge, this is the first large, multicentre study to provide detailed analysis of obstetric practice and outcome according to the presence of obstetric consultants on the labour ward.

## Methods

### Ethical Approval

Section 251 approval was granted by the Health Research Authority Confidentiality Advisory Group to process patient identifiable information without consent for the purposes of service evaluation. (CAG 2-06(a)/2013).

### Data Source

We used data extracted from the electronic maternity information systems (MIS) of 19 obstetric units across the UK that participated in the RCOG MIS Pilot Project. This project aimed to assess the feasibility of creating a national dataset using electronic patient records. The units were selected following a national call for participation from the RCOG. Ninety units responded positively and 25 were shortlisted on the basis of their size, geographic location, and type of MIS. HEK conducted follow-up telephone calls with the clinical director and data midwife at each unit to determine their ability to supply the required data item and their willingness to participate in the pilot. Following these telephone calls, 19 of the 25 hospitals confirmed that they were able to participate. Each hospital supplied a retrospective 12-mo extract of patient-level MIS data in accordance with a pre-defined specification (S1 Text). The extracts were pooled to create a single database comprising 112,458 infants born between 1 April 2012 and 31 March 2013, representing approximately 15% of the total number of births in the UK during this period.

The participating hospitals ranged in size from 1,800 to 9,800 deliveries per year. Two were large specialist women’s hospitals, 15 were teaching/university hospitals, and two were district general hospitals. Fifteen of the hospitals were located in England, one in Scotland, one in Wales, and two in Northern Ireland. All had an obstetric unit able to provide the full spectrum of obstetric care.

### Study Population

**Fig 1:**
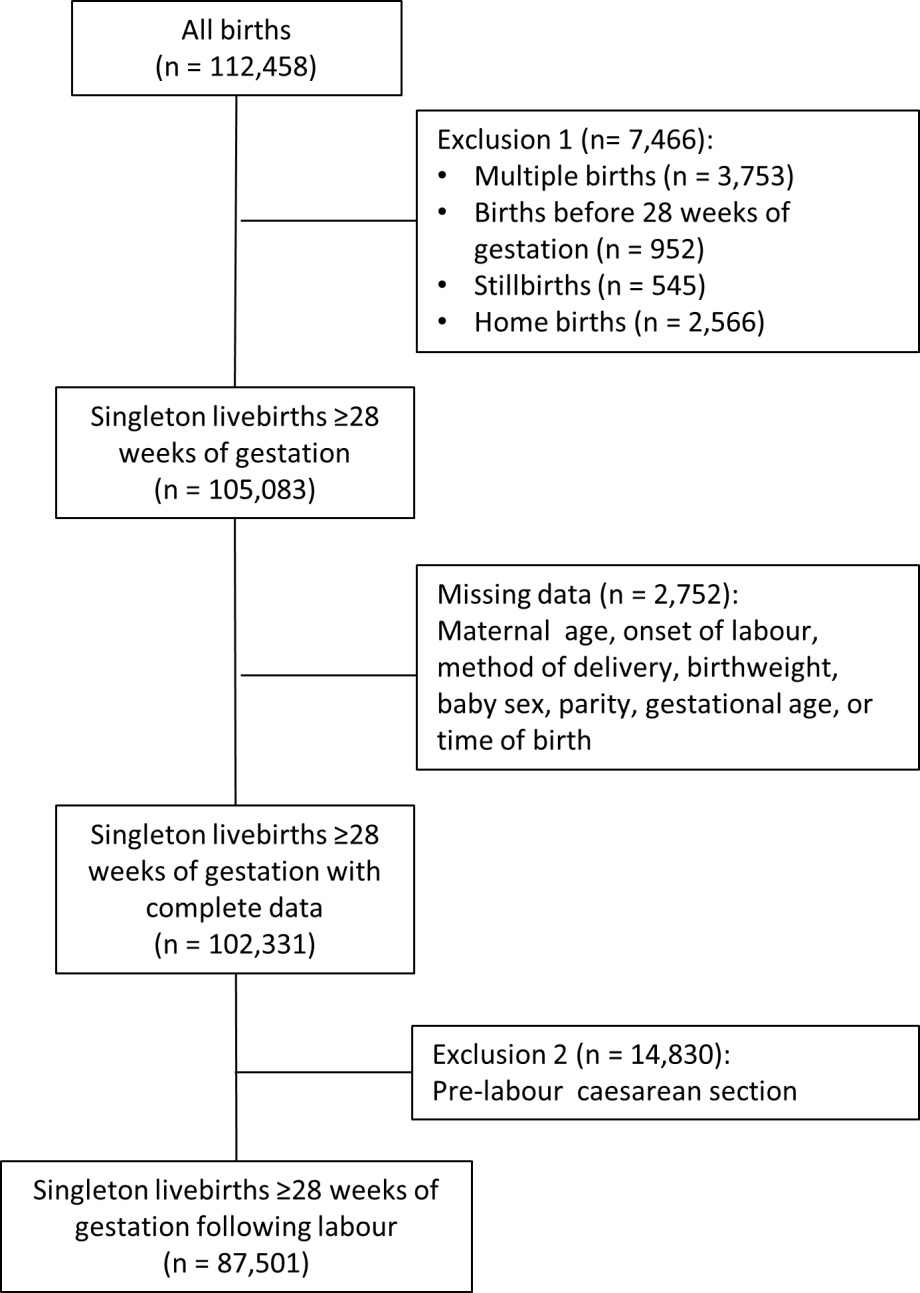
Selection of the cohort.

The records of women who had a singleton birth were extracted from the database, excluding deliveries before 28 completed weeks of gestation. We also excluded women who had a CS prior to the onset of labour because these are predominantly planned in advance and performed during normal working hours, carrying a low risk of neonatal death [16,17]. Hence, inclusion of such cases could lead to an over-estimate of the relative risk of adverse perinatal outcomes for “out-of-hours” deliveries. We could not assess the impact of consultant presence on perinatal mortality due to the rarity of the outcome and the size of the cohort. Moreover, we were not able to distinguish between antepartum and intrapartum stillbirths in the dataset. Antepartum deaths account for six in seven stillbirths [18] and in most cases occur some days prior to the delivery of the baby. The cohort was therefore restricted to livebirths (Fig 1). We did not exclude women with other co-morbidities or complicating risk factors.

The dataset included information on various maternal characteristics, including age, ethnicity, body mass index (BMI), and smoking status at booking. Obstetric risk factors included baby’s birthweight, gestational age at delivery, parity, previous CS, fetal presentation, baby’s sex, induction of labour, and mode of delivery.

“Consultant presence at time of birth” was defined as a binary variable by combining data on the time of birth with data from the consultant rotas for the period 2012–13 at each participating hospital. The rotas were obtained from the clinical directors of each maternity unit. Consultant presence refers to dedicated on-site labour ward cover, without other commitments such as antenatal or gynaecology clinics or theatre lists. For each day of the week, the time (in hours and minutes) that scheduled consultant presence on the labour ward began and ended was compared to the date and time of birth to define whether a delivery occurred within of a period of consultant presence (“in-hours”) or outside it (“out-of-hours”). The pattern of consultant presence across the week among the 19 hospitals is summarised in S1 Fig.

### Outcomes

Outcomes were selected to reflect different aspects of perinatal morbidity. Neonatal outcomes were measured using Apgar score < 7 at 5 min, umbilical cord pH < 7.1, and admission to neonatal care. Maternal outcomes were described by third or fourth degree perineal tear and PPH > 1,500ml, and the rates of intrapartum CS and instrumental delivery were used as indicators of obstetric activity. All hospitals collected Apgar score. Information on perineal tears, cord pH < 7.1, admission to neonatal care, and PPH was supplied by 18, 17, 16, and 15 hospitals, respectively.

### Statistical Analyses

We did not publish or pre-register a plan for this analysis. The analysis plan is described below, with any deviations noted in S2 Text.

We used proportions and medians to summarise the distribution of patient characteristics and the chi-square test and Kruskall-Wallis test for comparisons of dichotomous and continuous variables, respectively.

Multilevel multivariable logistic regression was used to estimate the crude and adjusted effects of consultant presence on the various outcomes, with the hospital of delivery modelled as a random-intercept. The potential confounding variables controlled for in all models were: maternal age (years), ethnicity (white, Asian, black, other, unknown), BMI (kg/m^2^), smoking status (smoker, non-smoker/ex-smoker, unknown), parity (0, ≥1), previous CS (yes, no), gestational age (completed weeks), fetal presentation (cephalic, non-cephalic), baby’s sex (male, female) and birthweight (g). For the continuous variables (maternal age, gestational age, and birthweight), quadratic terms were included in the models because there is clinical evidence that the relationship between these risk factors and the outcomes of interest is non-linear. Parity and previous CS were combined into one variable as these are not independent variables.

The completeness of data for the explanatory variables was generally good. No records were missing time of birth. Parity, mode of delivery, onset of labour, gestational age, birthweight, baby’s sex, and birth status were more than 99% complete. Patients missing one or more of these variables were dropped from the cohort (Fig 1). Ethnicity, BMI, and smoking status were missing in more than 1% of records, and we assigned missing values to a category of “unknown.” Apgar score was over 95% complete for all hospitals.

A sensitivity analyses limited to births at term (≥37 wk of gestation) was conducted to explore the possible risk of confounding due to preterm birth. Prematurity typically accounts for a significant proportion of adverse neonatal outcomes, and the inclusion of preterm deliveries could mask any out-of-hours effect observed among term deliveries. For example, a 30-wk infant will be admitted to neonatal care irrespective of the time of delivery or the care provided.

All statistical tests were two-sided and the level of significance was set at *p* < 0.05. All analyses were performed in Stata version 13 (StataCorp, College Station, TX, United States).

## Results

**Fig 2:**
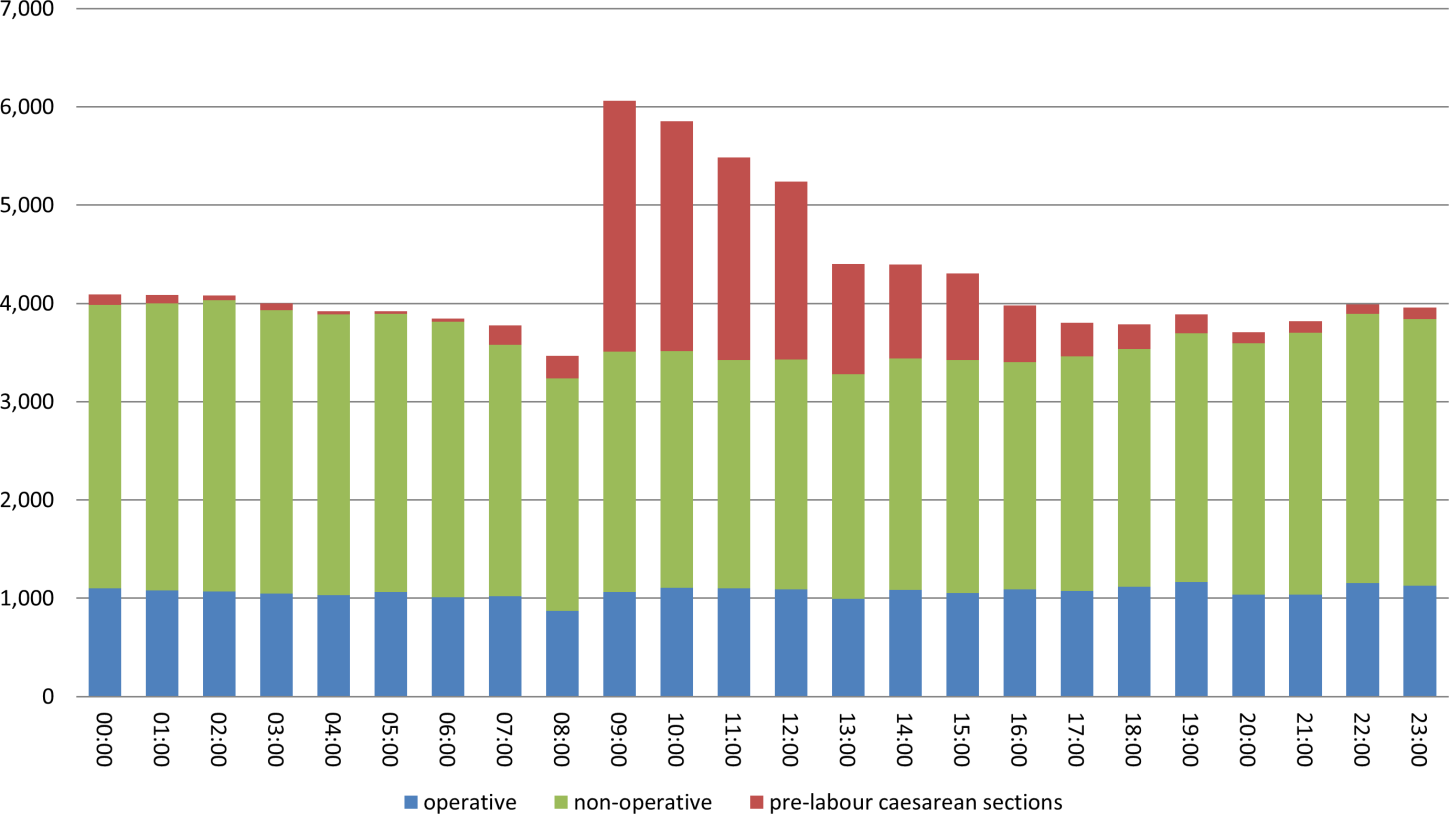
Number of births, by hour and mode of birth.

There were 112,458 deliveries in the sample between April 2012 and March 2013 (Fig 1). Restricting the cohort to singleton livebirths of at least 28 completed weeks of gestation excluded 7,466 records (6.6%), and dropping records with missing data in key explanatory variables removed a further 2,752 (2.4%). There was diurnal variation in the number of deliveries, with the majority of pre-labour CSs occurring between 9 a.m. and 7 p.m. (Fig 2). Included in the analysis were 87,501 deliveries following labour. Operative deliveries (intrapartum caesarean sections and instrumental deliveries) appeared to be evenly distributed throughout the day, with no evidence of a “spike” at the beginning and end of consultant shifts (Fig 2).

Consultant presence on the labour ward in the participating hospitals ranged from 51 h to 106 h per week. On weekdays, consultant presence generally began at 8:00 a.m. or 8:30 a.m. There was considerable variation among hospitals in the extent to which consultant presence extended into the evening, and in some units, consultants were rostered for a 24-h period on certain days (see S1 Fig). During weekends, consultant presence was typically only in the morning. The number of deliveries that occurred when a consultant was not rostered, i.e., out-of-hours, was 48,827 (55.8%).

Women who delivered out-of-hours shared similar demographic characteristics to women who delivered in-hours, with some small differences in maternal ethnicity, parity, and smoking status between the groups (Table 1). During in-hours, the rate of intrapartum CS and instrumental delivery were 13.4% and 17.0%, respectively, and 2.4% of women had a severe PPH and 3.6% experienced a severe perineal tear.

**Table 1:** Characteristics of the cohort, comparing in-hours with out-of-hours.

Characteristic	In-hours		Out-of-hours		*p*-Value
	N	Value	N	Value	
***Median (IQR) maternal age (years)***	38,674	30.0 (25.7–34.0)	48,827	30.1 (25.8–34.0)	0.267
***Ethnicity (%)***					<0.001
White	23,585	74.33	29,248	78.75	
Black	2,338	7.37	2,320	6.25	
Asian	3,438	10.83	3,199	8.61	
Other	2,370	7.47	2,375	6.39	
Missing^†^	6,942	-	11,685	-	
***BMI (%)***					0.084
<25	17,865	55.68	22,114	54.89	
25–30	9,082	28.29	11,519	28.61	
>30	5,143	16.03	6,647	16.50	
Missing^†^	6,583	-	8,548	-	
***Smoking status at booking (%)***					<0.001
Non-smoker	28,544	86.49	38,021	85.40	
Smoker	4,459	13.51	6,500	14.60	
Missing^†^	5,671	-	4,305	-	
***Parity (%)***					<0.001
Primiparous	19,552	50.56	23,168	47.45	
Multiparous, no previous CS	12,814	33.13	15,516	31.78	
Multiparous, previous CS	1,646	4.26	2,076	4.25	
Multiparous, unknown	4,661	12.05	8,067	16.52	
***Median (IQR) weeks gestation***	38,674	40 (39–41)	48,827	40 (39–41)	0.185
***Median (IQR) birthweight (g)***	38,674	3,400 (3,070–3,740)	48,827	3,400 (3,068–3,730)	0.225
***Fetal presentation (%)***					0.546
Cephalic	38,128	98.59	48,115	98.54	
Non-cephalic	545	1.41	712	1.46	

† Missing values are not included in the calculation of proportions.

After adjustment for maternal and obstetric risk factors, women who delivered out-of-hours were slightly less likely to have an intrapartum CS (OR 0.94; 95% CI 0.90 to 0.98) or an instrumental delivery (OR 0.92; 95% CI 0.89 to 0.96) than women who delivered in-hours (Table 2). There was no evidence of an overall difference in the incidence of severe PPH by consultant presence. There was weak evidence for a lower overall risk of perineal tears (OR 0.92; 95% CI 0.85 to 1.00).

**Table 2:** Crude and adjusted odds ratios for adverse perinatal outcomes, comparing in-hours and out-of-hours.

Overall cohort								
Outcome measures	In-hours		Out-of-hours		CrudeOR	Adjusted OR (95% CI)		*p*-Value
	N	Rate (%)	N	Rate (%)				
***Onset of labour/Mode of delivery***								
Intrapartum CS	38,674	13.43	48,827	12.72	0.92	0.93	(0.89 to 0.98)	0.003**
Instrumental delivery	38,674	16.97	48,827	15.61	0.91	0.92	(0.89 to 0.96)	<0.001***
***Maternal outcomes***								
Severe perineal tear (among vaginal deliveries)	30,788	3.58	39,967	3.27	0.90	0.92	(0.85 to 1.00)	0.054
Severe PPH (>1500ml)	30,858	2.38	36,094	2.31	1.01	1.03	(0.93 to 1.14)	0.589
***Neonatal outcomes***								
Apgar score < 7 at 5 min	38,384	1.25	47,206	1.33	1.06	1.06	(0.93 to 1.20)	0.374
Cord pH < 7.1	33,887	0.82	42,615	0.94	1.13	1.12	(0.96 to 1.31)	0.158
Admission to neonatal care	33,004	6.73	41,415	5.93	1.00	0.99	(0.93 to 1.06)	0.854

**Table 3:** Crude and adjusted odds ratios for adverse perinatal outcomes among term deliveries, comparing in-hours and out-of-hours.

Term deliveries (≥37 wk)								
Outcome measures	In-hours		Out-of-hours		Crude OR	Adjusted OR (95% CI)		*p*-Value
	N	Rate (%)	N	Rate (%)				
***Onset of labour/Mode of delivery***								
Intrapartum CS	36,826	13.12	46,541	12.41	0.92	0.94	(0.90 to 0.98)	0.005**
Instrumental delivery	36,826	17.19	46,541	15.83	0.91	0.92	(0.89 to 0.96)	<0.001***
***Maternal outcomes***								
Severe perineal tear (among vaginal deliveries)	29,423	3.66	38,220	3.36	0.90	0.92	(0.85 to 1.01)	0.072
Severe PPH (>1500ml)	29,383	2.40	34,419	2.35	1.02	1.04	(0.94 to 1.16)	0.451
***Neonatal outcomes***								
Apgar score < 7 at 5 min	36,602	1.13	45,063	1.16	1.03	1.03	(0.90 to 1.18)	0.650
Cord pH < 7.1	32,267	0.80	40,629	0.92	1.12	1.12	(0.95 to 1.32)	0.164
Admission to neonatal care	31,399	4.97	39,460	4.24	0.98	0.98	(0.91 to 1.05)	0.555

*p < 0.05

**p < 0.01

***p < 0.001

During in-hours, overall rates of Apgar score < 7 at 5 min, cord pH < 7.1 and admission to neonatal care were 1.25%, 0.82%, and 6.73%, respectively. We found no statistical association between the neonatal outcomes and consultant presence. There was no evidence of a difference in the rates of neonates with Apgar score < 7 at 5 min (OR 1.06; 95% CI 0.93 to 1.20), cord pH < 7.1 (OR 1.12; 95% CI 0.96 to 1.31) or admission to neonatal care (OR 0.99; 95% CI 0.93 to 1.06), after adjustment for maternal demographic and obstetric characteristics.

The restriction of the cohort to term deliveries (37 wk or later) in the sensitivity analysis did not alter the pattern of results observed in the cohort of all deliveries for neonatal outcomes, maternal outcomes, or obstetric interventions (Table 3).

## Discussion

This study analysed data from 19 UK obstetric units to investigate whether measures of neonatal and maternity morbidity varied during times when obstetric consultants were or were not present on the labour ward. Among women with singleton deliveries following labour, over half (55.8%) of all births occurred out-of-hours when consultants were not present on the labour ward. The birth rate peaked between 22:00 and 05:00.

Overall, we found no difference in the adjusted rates of morbidity among neonates born according to consultant presence on the three measures used in the study: Apgar score < 7 at 5 min, umbilical cord pH < 7.1, and admission to neonatal care. On the two measures of maternal morbidity, we found weak evidence that the adjusted rate of perineal tears was 10% lower during out-of-hours periods compared with in-hours, but there was no difference in adjusted rates of severePPH.

We also found that women who deliver out-of-hours were slightly less likely to have an obstetric intervention than women who delivered in-hours. One possible explanation for this finding is that in the absence of an urgent need for delivery, operative deliveries at the end of a night shift will tend to be deferred until the new team comes on, with consultant cover. There could be similar arguments made that at the end of a shift, teams may bring forward operative deliveries, not wanting to leave difficult deliveries until later.

The sensitivity analysis produced results that were broadly consistent with the results derived from the overall cohort. The restriction of the analysis to term infants had no material effect on the crude rates and the adjusted odds ratios.

There has been a broad consensus among medical professionals and policy makers that the duration of periods without consultant presence on the labour ward should decrease [6,11]. The policy stems from a series of studies that highlighted worse outcomes for babies born outside the normal weekdays. In particular, studies from other countries have reported an increase in asphyxia-related deaths among babies born at night [4,19–21]. In addition, figures from the UK National Patient Safety Agency showed that incidents of severe fetal compromise occurred more frequently between 8 p.m. and 4 a.m. [11].

### Comparison with Other Studies

Given the background of confidential enquiry reports highlighting that many cases of poor neonatal or maternal outcomes are linked to the failure to recognise and act on problems arising in labour [22–24], it might be expected that our study would show differences in outcomes during periods of time with and without consultant presence. Previous studies examining whether perinatal mortality and morbidity rates vary according to time of birth and have produced inconsistent findings. Some studies reported no difference [25–31], whereas others reported increased risks of mortality for births during the weekend [4,32–36], and/or the night [4,19–21,37,38]. A recent study reported some evidence of a “weekend effect” in perinatal mortality in England [36], although it was criticised for failing to exclude antepartum stillbirths from the outcome measure, leading to “unjustified extrapolations of what the results mean in terms of avoidable harm” [39].

That these differences are not apparent in this study may be due to various factors. Our study is smaller than those that are based on national data or use cohorts spanning several years and is therefore less likely to detect statistically significant differences in outcomes. Our study is also unique in that it uses a more nuanced exposure variable, which is likely to be a more accurate proxy of senior input than time of birth alone. As far as we are aware, no other multicentre studies have examined the extent to which variation in consultant presence on the labour ward is associated with maternal and neonatal outcomes.

Second, the results from several previous studies describe patterns of care among births that occurred during the 1980s and 1990s [4,19–21]. There have been considerable changes in the obstetric evidence base, diagnostic technology, and clinical governance since that time, which have improved the safety of NHS maternity services.

Third, since the mid-2000s, it has been recommended that UK obstetric units with over 2,500 deliveries annually have at least 40 h of consultant presence per week, and that larger units with over 5,000 annual deliveries have at least 60 h of consultant presence [11]. In their 2008 report, the Healthcare Commission reported that only 68% of English NHS trusts met the recommended standard. They also reported that roughly one in five midwives and one in four doctors thought more consultant obstetrician presence was needed on their labour ward [6]. The latest RCOG Workforce Census reported that by 2013, the mean number of hours of consultant presence on the labour ward in UK obstetric units had increased to 63.5 [12]. The 19 obstetric units that participated in this study had a mean of 75 h of consultant presence per week, which may be greater than in previous studies.

Finally, it is now expected that a consultant obstetrician should be available within 30 min outside the hours of consultant presence, and any risks associated with on-call cover may have changed [40].

### Methodological Considerations

A strength of this study is that it is based on a large, multicentre dataset of over 87,000 deliveries that occurred in 2012–13. It therefore provides a description of recent practice in the UK across a range of obstetric units. We excluded planned CS deliveries, as these carry a low risk of neonatal mortality and are predominantly carried out during “office hours.” Their inclusion could have led to an overestimate the risks of out-of-hours deliveries.

The study was also focused on severe morbidity, which has been suggested as a better indicator of the quality of intrapartum care in high-income countries than mortality [41], both for mothers [42–44] and neonates [45]. Mortality is now a rare complication of childbirth in high-income countries, and perinatal mortality rates in the UK are at their lowest recorded levels, at 6.0 per 1,000 live births [18]. Moreover, national data suggests that 86% of stillbirths involve death of the baby prior to the onset of labour, and the majority of neonatal deaths are due to anomalies and preterm birth, with intrapartum complications being an uncommon cause [18]. Low Apgar score has been shown to be strongly associated with the risk of neonatal and infant death ascribed to intrapartum hypoxia [46]. Cord pH < 7.1 is also an objective measure of fetal acidosis.

A weakness of observational studies is the potential for “bias by indication.” We minimised this risk by excluding multiple and very preterm infants with a high probability of an adverse outcome and, in that way, including a relatively homogenous group of deliveries. Furthermore, we also carried out a sensitivity analysis confined to term deliveries ≥37 wk. Finally, we risk-adjusted outcomes according to relevant maternal and obstetric risk factors.

Another limitation of the study was that we only had access to consultant rotas for each of the hospitals, which allowed us to calculate a hospital-specific out-of-hours classification of routine consultant presence. Consequently, the results are likely to be an underestimate of the effect of delivering out-of-hours compared to a study that would have information about consultant presence for each delivery. Serious complications arise for a minority of women and babies, and to detect the potentially small differences in outcomes that occur in childbirth, it might be necessary to have data on consultant presence at an individual-patient level. Other aspects of the organisation and delivery of maternity care, such as the availability and grade of other staff such as midwives, trainees, and healthcare assistants; patient triage protocols; or deviations from the rotas due to staff absence or vacancies may be important but were not investigated in this study. Some neonatal outcomes may also be related to non-obstetric medical staffing, such as availability of senior paediatricians. A related issue is that we only studied the association between selected outcomes and consultant presence at the time of birth. It is important to note that outcomes may also be influenced by whether or not a consultant is present earlier during labour, when crucial decisions are being made.

Finally, the hospitals in our study may not capture the full variation in obstetric care and outcomes in UK hospitals. The hospitals cover all of the geographic regions of the UK and include three district general hospitals with fewer than 5,000 deliveries per year. Nonetheless, most participating units were teaching or university hospitals with more than 5,000 deliveries per year, and all were selected from a list of hospitals who were able to provide electronic MIS data.

Taken together, the available evidence provides some reassurance that the organisation of maternity care in the UK allows for good planning and risk management. This suggests that politically driven efforts to target resources at increasing senior obstetricians attendance out-of-hours may not, in fact, lead to improved clinical outcomes for women and babies. However, there is a need for more robust national evidence on the quality of care delivered at all times of the week by maternity units employing different models of labour ward staffing. Ideally, studies should also consider longer-term outcomes, including cerebral palsy and school attainment.

## Supporting Information

S1 STROBE Checklist STROBE Checklist. (DOC)

S1 Fig Routine consultant presence on the labour ward according to hospital rotas. (TIF)

S1 Text RCOG MIS Pilot Project data specification for participating units. (PDF)

S2 Text Analysis history for the observational study described in this paper. (DOCX)

## Abbreviations

BMI: body mass index
CI: confidence interval
CS: caesarean section
MIS: maternity information systems
NHS: National Health Service
OR: odds ratio
PPH: postpartum haemorrhage
RCOG: Royal College of Obstetricians and Gynaecologists
