# Supplementary figures and images for: Birth “Out-of-Hours”: An Evaluation of Obstetric Practice and Outcome According to the Presence of Senior Obstetricians on the Labour Ward
Source: PLoS Med. 2016 Apr 19;13(4):e1002000. doi: 10.1371/journal.pmed.1002000 (PMC4836717; doi:10.1371/journal.pmed.1002000)

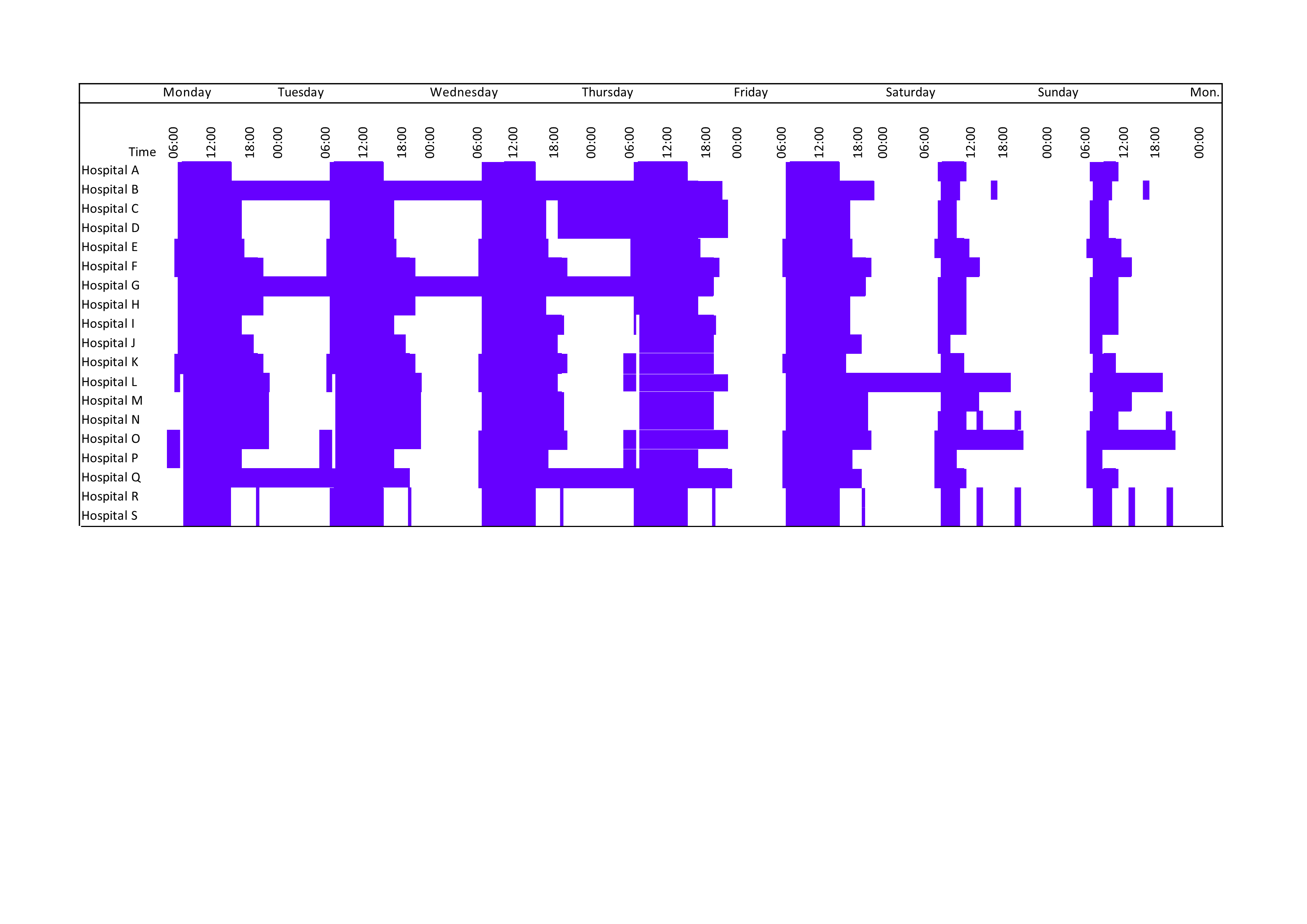

Supplement: S1 Fig — (TIF) [file pmed.1002000.s002.tif]
