# Supplementary material for: Birth “Out-of-Hours”: An Evaluation of Obstetric Practice and Outcome According to the Presence of Senior Obstetricians on the Labour Ward
Source: PLoS Med. 2016 Apr 19;13(4):e1002000. doi: 10.1371/journal.pmed.1002000 (PMC4836717; doi:10.1371/journal.pmed.1002000)
Supplement: S1 Text — (PDF) [file pmed.1002000.s003.pdf]

1. The following data items are required for all registerable births (including live births before 24 weeks of gestation and stillbirths after 24 weeks of gestation) that took place in your trust (including home births) between 1st April 2012 and 31st March 2013.

2. The data are required at the level of ONE ROW PER BABY (i.e. 2 rows for twins, 3 rows for triplets etc). In the case of multiple births, the maternal information (demographics, obstetric history, antenatal care) should be identical but neonatal information (e.g. mode of delivery, birth weight) may differ.

3. Preferred formats for the output are detailed below in Column C. These are largely based on national code definitions; however, if your system captures data in a different format or uses an alternative coding system, you do not need to re-code the data. Please simply send the raw data extract together with a data dictionary or similar to enable us to interpret the data.

4. If possible, please save the extract as a comma-separated value (CSV) file. For fields with multiple, non mutually-exclusive options (highlighted in green), please use a different delimiter (e.g. ; or |) to separate data items within a field.

5. The data extract must be transferred securely in an encrypted format as per the Data Sharing Agreement. Please contact Lynn Copley on 020 7869 6609 / lcopley@rcseng.ac.uk to discuss data transfer options.

6. If you have any queries regarding these instructions please contact Hannah Knight on 020 7772 6472 / hknight@rcog.org.uk

The preferred format of the data item is expressed in data type and length.

The data type is represented in either alphanumeric or numeric form. i.e.:

- an - indicating an alphanumeric data item
- n - indicating a numeric data item

The length is expressed in a numeric form e.g. 6, which would indicates a data item that captures 6 characters (Note - spaces are counted as characters).

In some cases, the length is proceeded with 'MAX' to indicate that the length is variable but has an upper limit. For instance a format of 'max
Examples of formats are:

- an2 - a data item in an alphanumeric format which captures 2 characters.
- max an2 - a data item in an alphanumeric format which captures a maximum of 2 characters.
- n3 - a data item in a numeric format which captures 3 numeric characters
- an7 - n:nnnnnnn - a data item in a alphanumeric format with specific data types for each character. Barring the second character, all characters are numeric. The second character is alphanumeric.
- n.nn - a data item in a numeric format which captures three numeric characters separated by a full stop (after the first character).

DATE and DATE TIME data items are in alphanumeric format, however, the format of these data items also explain the specific form of each character. i.e.:

- an10 - CCYY-MM-DD
  CCYY denotes the year, *MM* denotes the month and *DD* denotes the day in the month.

- an19 - YYYY-MM-DDThh:mm:ss
  YYYY denotes the year, *MM* denotes the month and *DD* denotes the day in the month.
  T is a prefixed value denoting that the subsequent characters relate to time (i.e. trusts will submit the letter *T*).
  hh denotes the hour, *mm* denotes the minute and *ss* denotes the second.

Data item	Description	Format
	**DEMOGRAPHICS**	
**Mother's NHS number**	The NHS Number of the mother in a maternity episode	n10 (nnnnnnnnnn)
**Postcode**	The postcode of usual address, as nominated by the mother	max an8
**Mother's date of birth**	Date of birth of the mother in a maternity episode	Preferred format: an10 (CCYY-MM-DD)
**Mother's ethnicity**	The ethnicity of the mother in a maternity episode as specified by herself	Preferred categories and codes listed below (based on 2001 census). If these are not the categories recorded in your MIS, please provide any relevant information needed to interpret your data.<br>**White**<br>A - White British<br>B - White Irish<br>C - Any other White background<br>**Mixed**<br>D - White and Black Caribbean<br>E - White and Black African<br>F - White Asian<br>G - Any other Mixed background<br>**Asian or Asian British**<br>H - Indian<br>J - Pakistani<br>K - Bangladeshi<br>L - Any other Asian Background<br>**Black or Black British**<br>M - Caribbean

		N - African<br>P - Any other Black background<br>**Other Ethnic Groups**<br>R - Chinese<br>S - Any other ethnic group<br>Z - Not Stated<br>99 - Not known
**Father's ethnicity**	The ethnicity of the father as specified by himself, or by the mother if the father is not present	Preferred categories and codes listed below (based on 2001 census). If these are not the categories recorded in your MIS, please provide any relevant information needed to interpret your data.<br>**White**<br>A - White British<br>B - White Irish<br>C - Any other White background<br>**Mixed**<br>D - White and Black Caribbean<br>E - White and Black African<br>F - White Asian<br>G - Any other Mixed background<br>**Asian or Asian British**<br>H - Indian<br>J - Pakistani<br>K - Bangladeshi<br>L - Any other Asian Background<br>**Black or Black British**<br>M - Caribbean<br>N - African<br>P - Any other Black background<br>**Other Ethnic Groups**<br>R - Chinese<br>S - Any other ethnic group<br>Z - Not Stated<br>99 - Not known

OBSTETRIC HISTORY

**Gravida**	Number of previous pregnancies (including miscarriages and abortions)	max n2
**Parity**	Number of previous registerable births (any birth >24 weeks of gestation, or with any signs of life)	max n2
**Caesarean section**	Has the mother delivered any previous baby by caesarean section?	Preferred format: N = no; Y = yes
**Instrumental delivery**	Were any of the woman's previous babies delivered with instrumental assistance?	Preferred format: N = no; Y = yes
**Preterm birth**	Were any of the woman's previous babies born before 37 completed weeks of gestation?	Preferred format: N = no; Y = yes
**Low birth weight**	Did any of the woman's previous babies weight less than 2500g at birth?	Preferred format: N = no; Y = yes
**Stillbirth**	Has the woman ever had a stillbirth (intrauterine fetal death after 24 completed weeks of gestation)?	Preferred format: N = no; Y = yes
**Pre-eclampsia, eclampsia**	Did the woman have preeclampsia or eclampsia during any previous pregnancy?	Preferred format: N = no; Y = yes
**Placenta accreta**	Did the woman have placenta acreta during any previous pregnancy?	Preferred format: N = no; Y = yes

ANTENATAL CARE

**Assisted conception**	Did the mother conceive through a method of assisted conception (e.g. IVF/IUI)	Preferred format: N = no; Y = yes
**Date of LMP**	Date of the last menstrual period as reported by the mother (if known)	Preferred format: an10 (CCYY-MM-DD)
**Gestation at booking**	Estimated gestational age at booking in days	max n3
**Maternal weight at booking**	The weight of the mother in kilograms at the Booking Appointment	maxn3.maxn3
**Maternal height**	The height of the mother in metres	n1.maxn2
**BMI at booking**	The body mass index of the mother at the Booking Appointment	n2.n1
**Smoking status at booking**	The mother's self-reported smoking status at the Booking Appointment	Preferred categories and codes listed below. If these are not the categories recorded in your MIS, please provide any relevant information needed to interpret your data.<br>01 - Current smoker<br>02 - Ex-smoker - Stopped after conception<br>03 - Ex-smoker - Stopped between conception and 12 months before conception<br>04 - Ex-smoker - Stopped more than 12 months before conception<br>05 - Non-smoker - history unknown<br>06 - Never smoked<br>09 - Unknown
**Alcohol (units per week)**	The typical number of units of alcohol the mother drinks, per week, as reported at the Booking Appointment	max n3
**Confirmed EDD**	The Estimated Date of Delivery, as agreed by ultrasound scan, LMP or Clinical Assessment	Preferred format: an10 (CCYY-MM-DD)
**Antenatal complications/diagnoses**	Any obstetric condition/s diagnosed in this pregnancy	Preferred categories and codes listed below. If these are not the categories recorded in your MIS, please provide any relevant information needed to interpret your data.<br>01 - Severe pre-eclampsia requiring pre-term birth<br>02 - Haemolytic anaemia, elevated liver enzymes and Low platelet count (HELLP)<br>03 - Eclampsia<br>05 - Liver cholestasis of pregnancy<br>06 - Gestational diabetes mellitus<br>07 - Gestational hypertension<br>08-Gestational proteinuria<br>09 - Antepartum haemorrhage<br>11 - Feto-maternal haemorrhage<br>18 - Symphysis pubis dysfunction<br>19 - Placenta praevia<br>20 - Severe pre-eclampsia
**Group B Streptococcus screening**	Was the mother screened for Group B Streptococcus?	Preferred format: N = no; Y = yes
**Pre-existing clinical conditions**	As identified at the Booking Appointment and based on the woman's past medical history, the diagnosis or type of diagnosis presenting a risk or complicating factor for this pregnancy	Preferred categories and codes listed below. If these are not the categories recorded in your MIS, please provide any relevant information needed to interpret your data.<br>01 - Hypertension<br>02 - Cardiac disease<br>03 - Renal disease<br>04 - Mental health disorder<br>05 - Thromboembolic disorder

		06 - Haematological disorder<br>07 - Central nervous system disorder<br>08 - Diabetes<br>09 - Autoimmune disease<br>10 - Cancer<br>12 - Infectious hepatitis A<br>13 - Serum Hepatitis B<br>14 - Hepatitis C<br>16 - Endocrine disorder<br>17 - Respiratory disease<br>18 - Gastrointestinal disorder<br>19 - Musculoskeletal disorder<br>20 - Gynaecological problems
**Intended delivery location**	Planned place of delivery (type)	Preferred categories and codes listed below. If these are not the categories recorded in your MIS, please provide any relevant information needed to interpret your data.<br>0 - In NHS hospital - delivery facilities associated with midwife ward<br>1 - At a domestic address<br>2 - In NHS hospital - delivery facilities associated with consultant ward<br>3 - In NHS hospital - delivery facilities associated with GMP ward<br>4 - In NHS hospital - delivery facilities associated with consultant/GMP/midwife ward inclusive of any combination of two of the professionals mentioned<br>5 - In private hospital<br>6 - In other hospital or institution<br>7 - In NHS hospital - ward or unit without delivery facilities<br>8 - None of the above<br>9 - Not known
**LABOUR AND DELIVERY**		
**Actual delivery location**	Location in which baby was delivered	Preferred categories and codes listed below. If these are not the categories recorded in your MIS, please provide any relevant information needed to interpret your data.<br>0 - In NHS hospital - delivery facilities associated with midwife ward<br>1 - At a domestic address<br>2 - In NHS hospital - delivery facilities associated with consultant ward<br>3 - In NHS hospital - delivery facilities associated with GMP ward<br>4 - In NHS hospital - delivery facilities associated with consultant/GMP/midwife ward inclusive of any combination of two of the professionals mentioned<br>5 - In private hospital<br>6 - In other hospital or institution<br>7 - In NHS hospital - ward or unit without delivery facilities<br>8 - None of the above<br>9 - Not known
**Transferred in**	Was the woman transferred to this unit for her antenatal care, labour or delivery (as opposed to booking at this hospital)?	Preferred format: N = no; Y = yes
**Smoking status at delivery**	The mother's self-reported smoking status at delivery	Preferred categories and codes listed below. If these are not the categories recorded in your MIS, please provide any relevant information needed to interpret your data.<br>01 - Current smoker<br>02 - Ex-smoker - Stopped after conception<br>03 - Ex-smoker - Stopped between conception and 12 months before conception<br>04 - Ex-smoker - Stopped more than 12 months before conception<br>05 - Non-smoker - history unknown<br>06 - Never smoked<br>09 - Unknown
**Number of infants this delivery**	Number of registerable infants delivered	n1
**Onset of labour**	The method used to induce (initiate) labour, rather than to accelerate it.	Preferred categories and codes listed below. If these are not the categories recorded in your MIS, please provide any relevant information needed to interpret your data.<br>1 - Spontaneous: the onset of regular contractions whether or not preceded by spontaneous rupture of the membranes<br>2 - Not applicable: caesarean section carried out prior to onset of labour or immediately following the onset of labour, when the decision was made before labour<br>3 - Surgical induction by amniotomy<br>4 - Medical induction, including the administration of agents either orally, intravenously or intravaginally with the intention of initiating labour<br>5 = Combination of surgical induction and medical induction<br>9 = Not known
**Type of medical induction (if applicable i.e. Option 4 in the previous question)**	The agent used for medical induction of labour	Preferred categories and codes listed below. If these are not the categories recorded in your MIS, please provide any relevant information needed to interpret your data.<br>01 - Mifepristone<br>02 - Misoprostol<br>03 - Prostaglandin<br>04 - Oxytocin<br>05 - Unknown
**Labour augmentation**		Preferred format: N = no; Y = yes
**Time of onset of established labour**	Date/time when established labour is confirmed - regular painful contractions and progressive cervical dilatation	Preferred format: an19 YYYY-MM-DDThh:mm:ss
**Time of onset of second stage**	Signs or evidence of full dilatation of cervix	Preferred format: an19 YYYY-MM-DDThh:mm:ss
**Anaesthesia in labour and delivery**	Type of anaesthesia used within the labour & delivery episode	Preferred categories and codes listed below. If these are not the categories recorded in your MIS, please provide any relevant information needed to interpret your data.<br>01 - General anaesthetic<br>02 - Epidural or caudal anaesthetic<br>03 - Spinal anaesthetic<br>09 - Pudendal block anaesthetic<br>97 - Other anaesthetic or analgesic only<br>98 - No anaesthetic administered
**ECV before labour**	Was external cephalic version performed before the onset of labour?	Preferred format: N = no; Y = yes
**Lead maternity care professional**	The professional category of the clinician with overall responsibility for care during the pregnancy	Preferred categories and codes listed below. If these are not the categories recorded in your MIS, please provide any relevant information needed to interpret your data.<br>060 - Consultant Obstetrician<br>160 - General medical practitioner<br>170 - Midwife
**Senior person present at delivery**	The professional category of the most senior clinician present during the delivery	Preferred categories and codes listed below. If these are not the categories recorded in your MIS, please provide any relevant information needed to interpret your data.<br>060 - Consultant Obstetrician<br>160 - General medical practitioner<br>170 - Midwife
**Presentation at onset of labour/delivery**	The presentation of the fetus at onset of labour/delivery	Preferred categories and codes listed below. If these are not the categories recorded in your MIS, please provide any relevant information needed to interpret your data.<br>01 - Cephalic<br>02 - Breech<br>03 - Transverse/oblique<br>04 - Not known<br>XX - Other

**Method of delivery**	The method for delivering baby	Preferred categories and codes listed below. If these are not the categories recorded in your MIS, please provide any relevant information needed to interpret your data.<br>0 - Spontaneous Vertex<br>1 - Spontaneous Other Cephalic<br>2 - Low forceps, not breech<br>3 - Other Forceps, not breech<br>4 - Ventouse, Vacuum extraction<br>5 - Breech<br>6 - Breech Extraction<br>7 - Elective caesarean section<br>8 - Emergency caesarean section<br>9 - Other
**Perineal tears**	Whether or not there was a traumatic lesion of the genital tract	Preferred categories and codes listed below. If these are not the categories recorded in your MIS, please provide any relevant information needed to interpret your data.<br>01 - None<br>02 - Labial tear<br>03 - Vaginal wall tear<br>04 - Perineal tear - first degree<br>05 - Perineal tear - second degree<br>06 - Perineal tear - third degree<br>07 - Perineal tear - fourth degree<br>09 - Cervical tear<br>10 - Urethral tear<br>11 - Clitoral tear<br>12 - Anterior incision
**Episiotomy**	Whether or not an episiotomy was performed	Preferred format: N = no; Y = yes
**Maternal critical incident**	Instance of a critical incident occurring	Preferred categories and codes listed below. If these are not the categories recorded in your MIS, please provide any relevant information needed to interpret your data.<br>01 - Undiagnosed breech<br>02 -PPH >=500ml and <=999ml<br>03 - PPH >= 1000ml and <=1499ml<br>04 - PPH >= 1500ml<br>05 - Return to theatre<br>06 - Hysterectomy / laparotomy<br>07 - Anaesthetic complications<br>08 - Intensive care admission<br>09 - Venous thromboembolism<br>10 - Pulmonary embolism<br>11 - Unsuccessful forceps or ventouse
**Date and time of birth**	Date and time of birth of the baby	Preferred format: an19 YYYY-MM-DDThh:mm:ss
**Delivery outcome**	Outcome of delivery	Preferred categories and codes listed below. If these are not the categories recorded in your MIS, please provide any relevant information needed to interpret your data.<br>10 - Live birth<br>20 - Stillbirth<br>30 - Miscarriage<br>40 - Termination of Pregnancy < 24weeks<br>50 - Termination of Pregnancy >= 24weeks<br>XX - Other inc vanishing/papyraceous twin, ectopic
**Birth weight**	Weight of the baby at birth in grams	max n4
**Gestational age**	Gestation at date of birth in days	max n3
**Birth order**	Sequence in which the baby was born (if multiple)	n1
**Sex of baby**	Sex of the baby	Preferred categories and codes listed below. If these are not the categories recorded in your MIS, please provide any relevant information needed to interpret your data.<br>0 - Not Known (not recorded)<br>1 - Male<br>2 - Female<br>9 - Not Specified
**Cord blood gases**	pH of umbilical (venous) blood sample	n.n2
**Apgar score at 1 minute**	The Apgar score of the neonate 1 minute after delivery	max n2
**Apgar score at 5 minutes**	The Apgar score of the neonate 5 minutes after delivery	max n2
**Apgar score at 10 minutes**	The Apgar score of the neonate 10 minutes after delivery	max n2
**Baby's NHS number**	The NHS Number of the baby	n10 (nnnnnnnnnn)
**Neonatal procedures/diagnoses**	A neonatal diagnosis, as captured to the point of the baby's discharge from maternity services or neonatal services	Preferred categories and codes listed below. If these are not the categories recorded in your MIS, please provide any relevant information needed to interpret your data.<br>01 - Shoulder dystocia<br>02 - Cord prolapse<br>03 - Acute fetal compromise<br>04 - Fetal acidaemia<br>05 - Meconium Aspiration Syndrome<br>06 - Acute blood loss<br>07 - Jaundice requiring phototherapy<br>08 - Erb's Palsy<br>09 - Neonatal abstinence syndrome<br>10 - Birth trauma to the newborn<br>11 - Fetal laceration at caesarean section<br>12 - Cord pH < 7.1 venous<br>13 - Neonatal seizures<br>14 - Undiagnosed fetal abnormality<br>15 - European Congenital Anomalies or Twins (Eurocat)
**Date admitted to NICU/SCBU, if applicable**	Date/time on which baby was admitted to Neonatal Unit (NNU)	Preferred format: YYYY-MM-DDThh:mm:ss
**Antibiotic treatment for Group B Streptococcus**	Was antibiotic treatment given to the neonate for Group B Streptococcus?	Preferred format: N = no; Y = yes
	**DISCHARGE**	
**Maternal Death**	Date/time of death of mother during the antenatal, intrapartum and postpartum periods. The postpartum period only covers death to the point the woman gets discharged from maternity services.	Preferred format: YYYY-MM-DDThh:mm:ss
**Neonatal Death**	Date and time of death of baby, before 28 completed days of birth	Preferred format: YYYY-MM-DDThh:mm:ss
**Date of maternal discharge**	Date that the mother is discharged home	Preferred format: an10 (CCYY-MM-DD)
**Date of neonatal discharge**	Date that the neonate is discharged home	Preferred format: an10 (CCYY-MM-DD)
