# Supplementary material for: Birth “Out-of-Hours”: An Evaluation of Obstetric Practice and Outcome According to the Presence of Senior Obstetricians on the Labour Ward
Source: PLoS Med. 2016 Apr 19;13(4):e1002000. doi: 10.1371/journal.pmed.1002000 (PMC4836717; doi:10.1371/journal.pmed.1002000)
Supplement: S2 Text — (DOCX) [file pmed.1002000.s004.docx]

S1 Text. Analysis history for the observational study described in: Knight HE, van der Meulen JH, Gurol-Urganci I, Smith GCS, Kiran A, Thornton S, Richmond D, Cameron A, Cromwell DA. Birth 'out-of-hours': an evaluation of obstetric practice and outcome according to the presence of senior obstetricians on the labour ward. PLOS Medicine.

We did not publish or pre-register a protocol for this secondary analysis of data from maternity unit information systems (MIS). We followed a clear analysis plan, as described in the methods section. Further details on the analysis history are described below:

1. The study was motivated by the question “what is the variation in obstetric practice and outcome according to the presence of obstetric consultants on the labour ward?” which addressed some of the limitations of previously published studies on this topic.
2. The inclusion/exclusion criteria for the study were established at the outset of the study. As described in the manuscript, we extracted the records of women who had a singleton birth from the database, excluding deliveries before 28 completed weeks of gestation. We also excluded women who had a caesarean section prior to labour. We originally intended to include stillbirths in the cohort because we planned to examine intrapartum stillbirth as one of the neonatal outcomes in our analysis. However, as explained in the Methods, the data collected from the MIS databases did not enable us to differentiate between antepartum and intrapartum stillbirth. Antepartum deaths account for 6 in 7 stillbirths [18] and in most cases occur some days prior to the delivery of the baby. The cohort was therefore restricted to livebirths.
3. The statistical approach was determined at the outset and was not changed. As described in the manuscript, we conducted sensitivity analyses to interrogate the robustness of our findings. The sensitivity analyses were limited to births at term (≥37 weeks of gestation) in order to explore the possible risk of confounding due to preterm birth. Prematurity typically accounts for a significant proportion of adverse neonatal outcomes and the inclusion of preterm deliveries could have masked any ‘out-of-hours’ effect observed among term deliveries. For example, a 30-week infant will be admitted to neonatal care irrespective of the time of delivery or the care provided.

We also conducted an exploratory subgroup analysis to test for potential bias by indication. The analysis used four mutually exclusive subgroups of deliveries based on type of labour onset (spontaneous or not) and mode of delivery (operative delivery or not). These subgroups were selected because they may give an insight into specific processes of care and were defined a priori. As described in the paper by Gijsen et al [5], induction of labour and caesarean section can influence the time of birth of a high-risk pregnancy. We chose not to report the findings of this analysis in the manuscript because the pattern of results from the subgroup analysis was consistent with the overall findings.

1. Following feedback from the statistical reviewer, we made a minor amendment to the way that some variables were included in the multivariable logistic regression models. For continuous variables, quadratic terms were added to the models because there is clinical evidence that the relationship between these risk factors and the outcomes of interest is non-linear. This amendment has had no significant impact on the results.
